# Detectability of Breast Tumor by a Hand-held Impulse-Radar Detector: Performance Evaluation and Pilot Clinical Study

**DOI:** 10.1038/s41598-017-16617-6

**Published:** 2017-11-27

**Authors:** Hang Song, Shinsuke Sasada, Takayuki Kadoya, Morihito Okada, Koji Arihiro, Xia Xiao, Takamaro Kikkawa

**Affiliations:** 1 Research Institute for Nanodevice and Bio Systems, Hiroshima University, 1-4-2 Kagamiyama, Higashi-hiroshima, Hiroshima, 739-8527 Japan; 2 Department of Breast Surgery, Hiroshima University Hospital, Hiroshima University, Hiroshima, Japan; 3 Department of Surgical Oncology, Research Institute for Radiation Biology and Medicine, Hiroshima University, Hiroshima, Japan; 4 Department of Pathology, Hiroshima University Hospital, Hiroshima University, Hiroshima, Japan; 5 Tianjin Key Laboratory of Imaging and Sensing Microelectronic Technology, School of Microelectronics, Tianjin University, Tianjin, 300072 China

## Abstract

In this report, a hand-held impulse-radar breast cancer detector is presented and the detectability of malignant breast tumors is demonstrated in the clinical test at Hiroshima University Hospital, Hiroshima, Japan. The core functional parts of the detector consist of 65-nm technology complementary metal-oxide-semiconductor (CMOS) integrated circuits covering the ultrawideband width from 3.1 to 10.6 GHz, which enable the generation and transmission of Gaussian monocycle pulse (GMP) with the pulse width of 160 ps and single port eight throw (SP8T) switching matrices for controlling the combination of 4 × 4 cross-shaped dome antenna array. The detector is designed to be placed on the breast with the patient in the supine position. The detectability of malignant tumors is confirmed in excised breast tissues after total mastectomy surgery. The three-dimensional positions of the tumors in the imaging results are consistent with the results of histopathology analysis. The clinical tests are conducted by a clinical doctor for five patients at the hospital. The malignant tumors include invasive ductal carcinoma (IDC) and ductal carcinoma in situ (DCIS). The final confocal imaging results are consistent with those of Magnetic Resonance Imaging (MRI), demonstrating the feasibility of the hand-held impulse-radar detector for malignant breast tumors.

## Introduction

In clinical diagnosis, X-ray mammography is one of the most commonly used techniques for mass health examinations of breast cancers. However, the ionizing radiation is a fatal drawback which will limit the use for cancer detection and frequent breast screening. Therefore, non-radiation technique with easy access for regular breast health assessment is highly desired. In recent comprehensive studies on the electrical properties of the breast tissues, it is revealed that the electrical properties of the breast cancer are different from those of normal tissues^1–3^. Based on the fact, the microwave breast imaging has been proposed and studied, mainly for breast cancer detection, breast health monitoring, breast tumor screening and chemotherapy monitoring^4–11^. Since the microwave has no ionizing radiation, it is considered to be a promising alternative to the X-ray mammography.

Generally, two kinds of microwave imaging methods are used for different goals. On one hand, the microwave tomography aims at mapping the dielectric distributions of the whole breast^12–14^. On the other hand, the radar-based method mainly aims at reconstructing the position of the strong reflection from the breast interior^15–19^. Recent complete imaging systems for clinical application reflect the state-of-the-art of this technology. Meaney *et al*. have developed a microwave tomography system, employing an array of 16 modified monopole antennas^20^. Another version using rotating bi-static angle-bent antennas was also proposed^21^. In the system, the patient lies in the prone position and the antennas are oriented in the vertical position around the breast to emit and receive monochromatic microwaves. Meanwhile, the simulated signals from a numerical model are calculated using a forward solver. By comparing the measured data with the simulated counterparts, the properties of the breast are estimated. The system has been applied to breast imaging and chemotherapy monitoring and initial clinical results were reported^22^. Klemm *et al*. have developed a multi-static radar-based detection system, employing ultrawideband (UWB) antenna array^23–25^. The antennas are mounted in a hemispherical arrangement. During the detection, the synthesized short pulse is formed based on step frequency continuous wave (SFCW) method using a vector network analyzer (VNA) and the antennas are stimulated in turn. The reflections are collected and the modified delay-and-sum (DAS) algorithm is used to create the breast image to identify the target position. Clinical evaluation of the prototype system has been reported and successful detection of the breast tumor was demonstrated^26^. Fear *et al*. have developed a mono-static radar-based system, employing a single Vivaldi antenna with director^27,28^. The antenna is attached to a movable arm. A laser is used to identify the breast contour^29^. Signals are recorded by changing the antenna position and similar to the multi-static approach, the DAS algorithm is used for image generation. This system has been applied to several patients and encouraging results were reported^30^. Porter *et al*. have developed a time-domain radar system, employing antenna array for daily breast health monitoring^31,32^. The system employs time-domain signals generated from a commercial pulse signal generator. From the time flight of the signal, the relative permittivity of the breast is estimated. By monitoring the change of the permittivity, the risk of getting cancer is forecasted. Clinical studies have been carried out on healthy volunteer breasts and the fluctuation of the breast dielectric properties was demonstrated and analyzed^33^.

The prototypes mentioned above depend on some commercial heavy equipment, resulting in lack of portability. For the easy access of individual user, the minimization is an important issue. Many groups are engaged in developing compact microwave imaging systems^34–36^. However, there are few test reports about the clinical applications of the compact systems. In our previous work, we have developed the essential modules for microwave imaging. They are the pulse generator, switching matrix and sampling module using complementary metal-oxide-semiconductor (CMOS) integrated circuits^37–41^. Owing to these modules, compact radar-based imaging system prototypes were constructed^42,43^.

In this report, we present a new hand-held version of the prototype detector aiming at clinical use. In this new prototype, a cross-shaped dome antenna array is designed for covering human breast enabling application in clinical trials. The circuits are redesigned to adapt to the rotation feature. The automatic and precise rotation measurement is achieved by introducing a step motor. The resolution of the sampled signal is dramatically enhanced by utilizing a 12-bit analog to digital converter (ADC) compared with the 4-bit accuracy in the previous prototypes. Comparing with the other prototype systems based on heavy equipment, this detector is compact, portable and allows easy access to everyone. The performance of the detector is verified by phantoms and the initial clinical test results are reported. To the best of our knowledge, this is the first report of the clinical trial results using compact detection system.

## System composition

### Architecture

**Figure 1:**
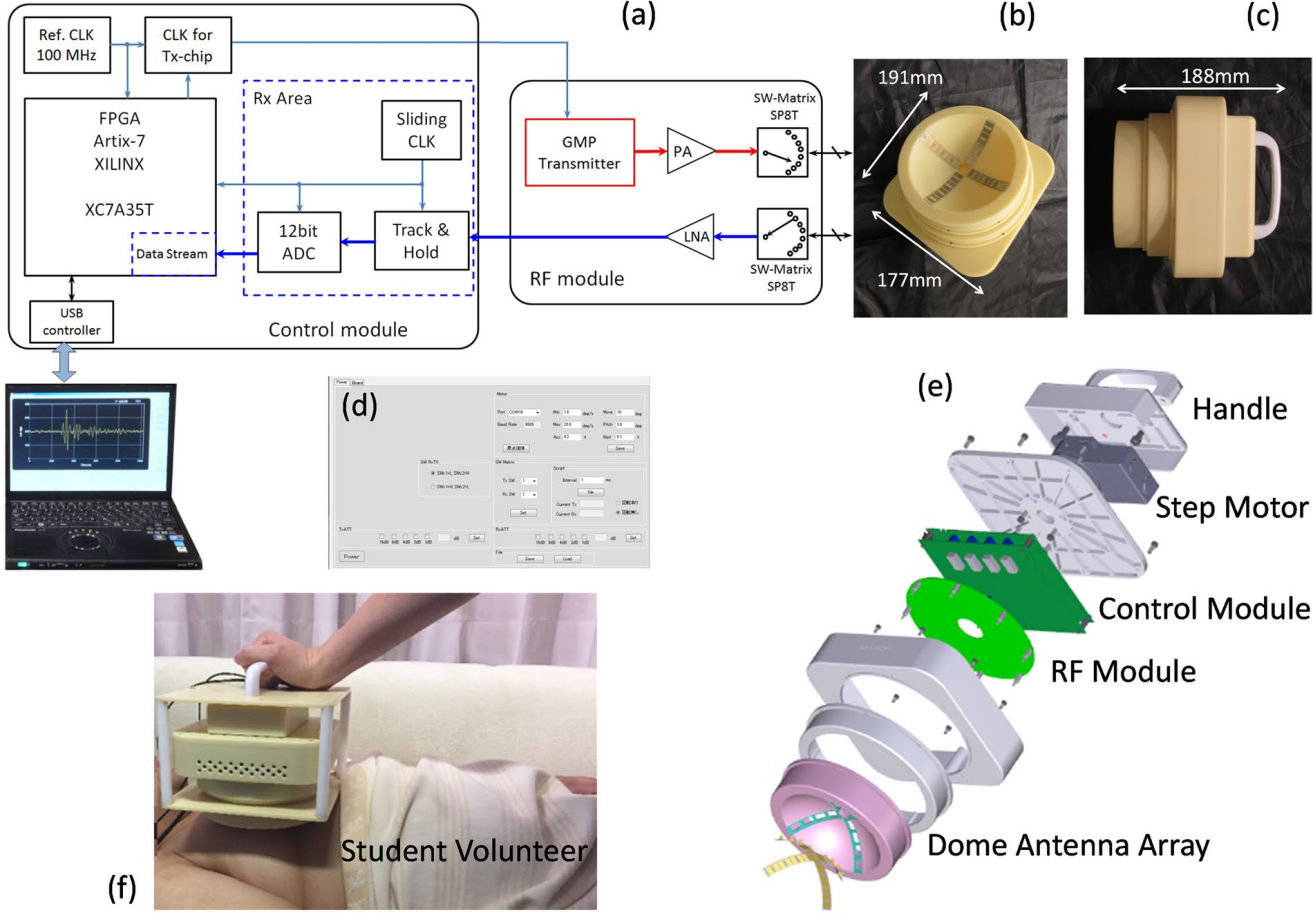
The architecture of the breast cancer detector. (**a**) The block diagram of the circuits. (**b**) Photograph of the bottom view showing the dome antenna array. (**c**) Photograph of the side view. (**d**) The control software graphic interface. (**e**) The breakdown diagram of the system. (**f**) The hand-held microwave breast cancer detector system in position for electrical testing. (Photo used with the permission of the student volunteer).

The architecture of the hand-held breast cancer detector is depicted in Fig. 1. The total size of the detector is 19.1 cm × 17.7 cm × 18.8 cm. This detector consists of a handle, a step-motor, a control module, a radio-frequency (RF) module and a dome antenna array. The block diagram of the circuits is shown in Fig. 1(a). The dome antenna array is connected with the circuits through the two single-pole-8-throw (SP8T) switching matrices (SW). The antenna configuration is shown in Fig. 1(b), in which a cross-shape 4 × 4 antenna array is formed. The step motor is mounted on the top of the detector as shown in Fig. 1(e). The step-motor can rotate with 1 degree/step accuracy. The detector is connected to a laptop computer by a USB cable for exchanging data and sending command. The customized software is developed to control the operation of the detector. The graphic interface of the software is shown in Fig. 1(d). In the clinical applications, a plastic cover is installed on the antenna dome to protect patient and mitigate friction during rotation. The patients are asked to lie in the supine position and the detector is put on the breast as shown in Fig. 1(f).

### Circuit design

**Figure 2:**
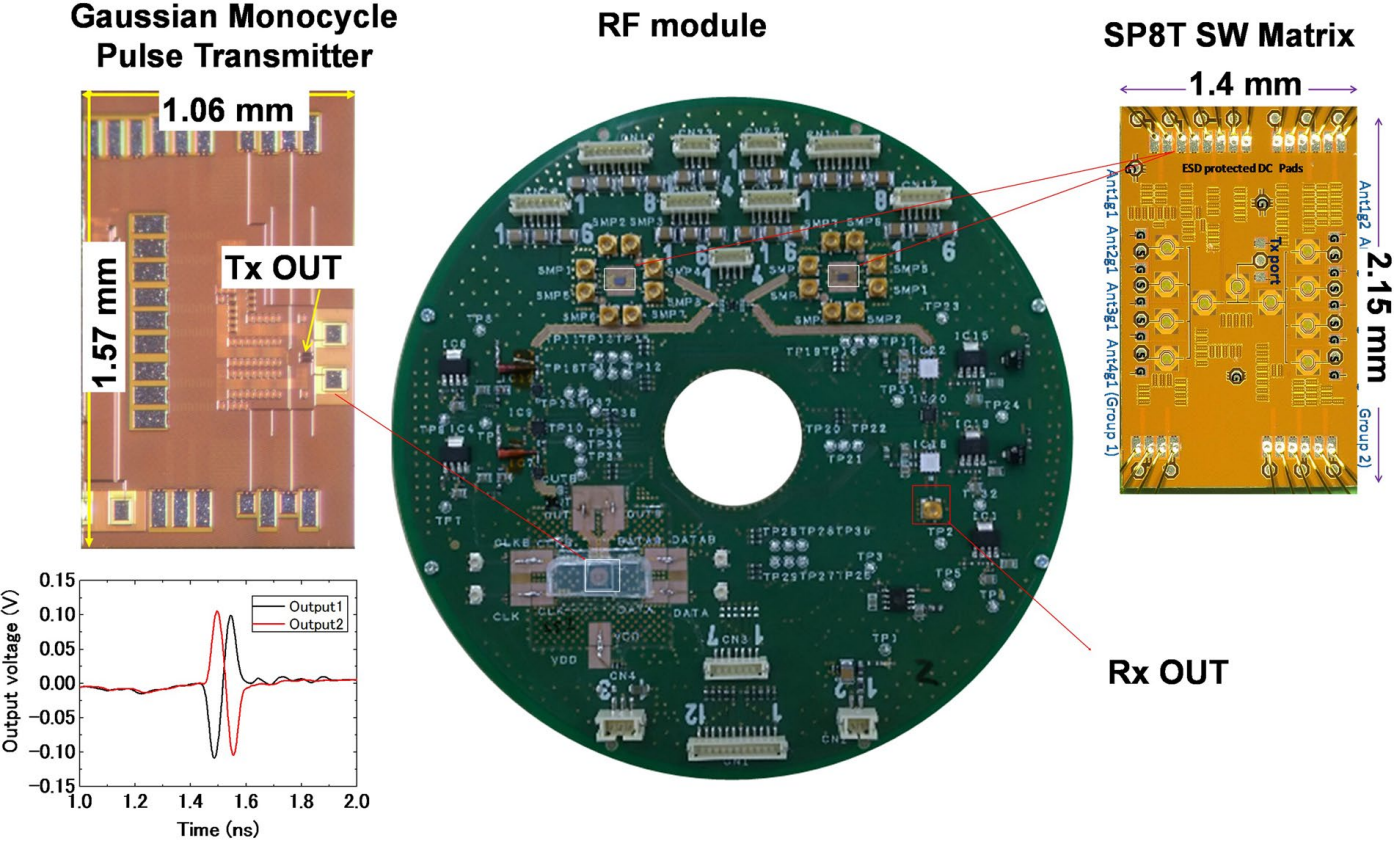
Photographs of the RF module and CMOS integrated circuit chips with the generated GMP waveforms.

The Gaussian monocycle pulse (GMP) generator^37,38^, two single-pole-eight-throw (SP8T) switching matrix (SW) modules and the amplifier module are integrated into the RF module. The Tx SP8T-SW controls 8 transmitting antennas and the Rx SP8T-SW controls 8 receiving antennas^39^. The RF module is fabricated on a disk-like printed circuit board (PCB) to adapt to the rotation feature as shown in Fig. 2. The GMP transmitter chip is assembled on the PCB and connected by wire bonding. Two SP8T SW chips are assembled by flip-chip bonding and connected to 16 mini SMP connectors through microstrip lines. The GMP transmitter generates differential waveforms from the two output ports as shown in Fig. 2. The received signals from array antennas are transmitted through the Rx SP8T-SW to the Rx module through Rx OUT. The receiving (Rx) module is integrated into the control module as shown in Fig. 1(a). It mainly consists of a track and hold (HMC760LC4B), a 12bit ADC (AD9233), and a differential amplifier (AD8352).

### Antenna design

**Figure 3:**
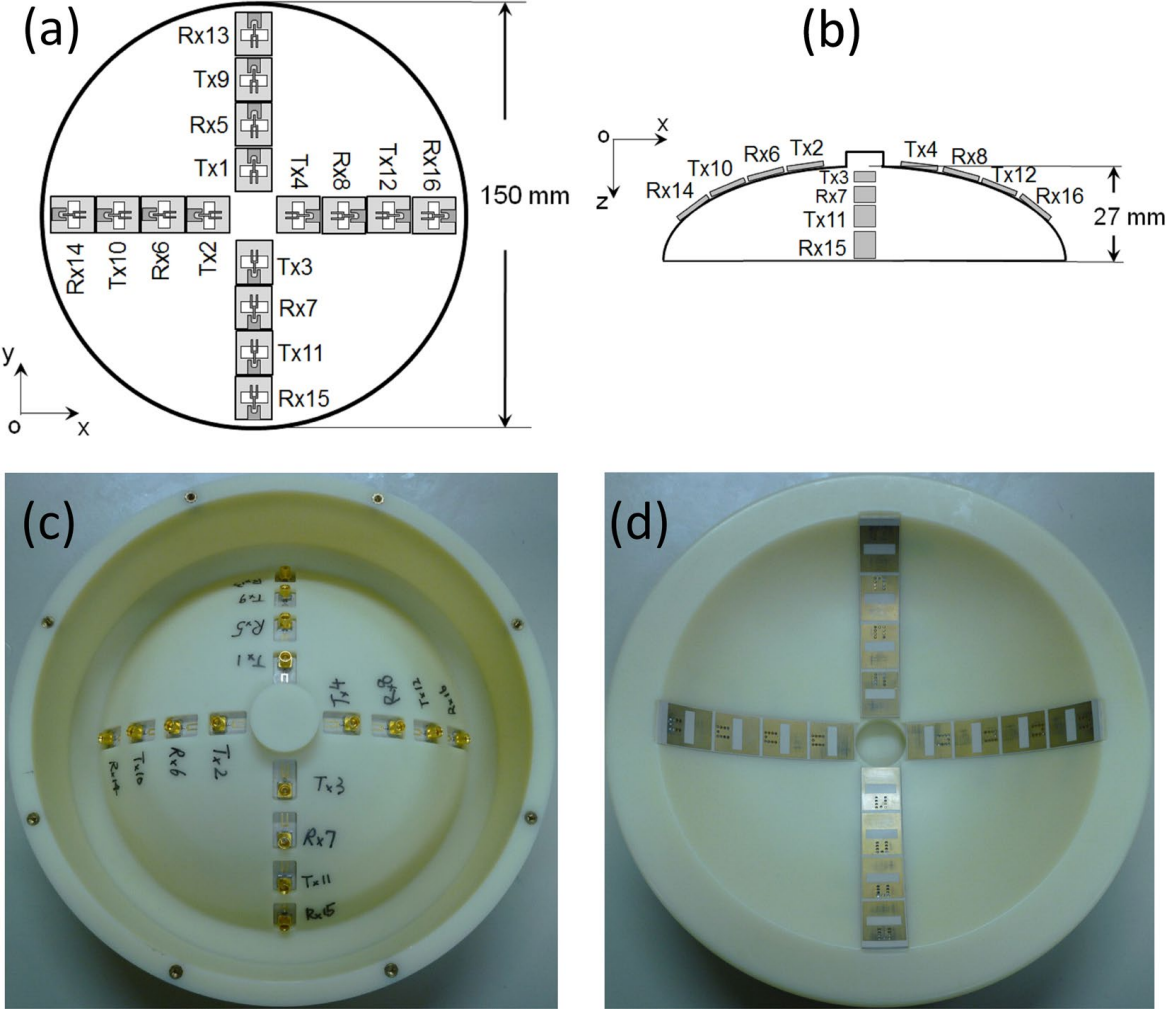
Dome antenna array design. (**a**) The top view of the antenna in *x-y* plane. (**b**) The side view of the antenna in *x-z* plane. (**c**) Top view photograph. (**d**) Bottom view photograph.

The dome antenna array consists of 16 elements and is connected to switching matrix by coaxial cables. The antenna element is a planar slot UWB (ultrawideband) antenna with the size of 11 mm × 13.1 mm × 0.635 mm^41^. The substrate of the antenna is RT/duroid 6010 with the dielectric constant of 10.2. The array is formed by 4 orthogonal antenna lines in a symmetrical cross shape as shown in Fig. 3. Each column has 4 element antennas which are fabricated on the substrate with a 1-mm space between each antenna. In each column, the first and third antennas are used as the transmitters (Tx) and the second and fourth antennas are the receivers (Rx). The positions of Tx1~Tx4 in z axis are the same. The other 3 groups, Rx5~Rx8 group, Tx9~Rx12 group and Rx13~Rx16 group have the same z positions, respectively. In the detector, two SP8T (single-pole-8-throw) switching matrices are utilized to control the transmitting route and receiving route, respectively. Therefore, the 16 antennas are divided into two groups. Each group has 8 antennas. The combinations are selected to cover more space to obtain adequate reflected information. As shown in ref.^39^, the output port on each switching matrix has the same performance. Therefore, the switching sequence is as follows. The Tx port is selected first, and then the Rx ports are selected in turn from 1 to 8. Next, the Tx port is changed and the Rx ports are selected successively. A dome shell is made of ABS (Acrylonitrile butadiene styrene) to hold the antennas. 4 line notches are engraved on the inner side of the shell. The 4 antenna columns are inserted into the notches with slightly bending and consolidated using a super glue. On the surface of the shell, 16 holes are drilled to let the connector go through. The radius of the inner dome curvature is 76 mm. At the center of the dome, there is a deep hole to hold the nipple for clinical experiment. The antennas are designed for placing on the skin of the breast without air gap^41^ and the influence of the casing materials is taken into consideration to reduce the impedance mismatching. Therefore, the coupling medium, which is used in other systems, is not needed for our prototype. In our detector, the glycerin is put in the narrow gap between the cover and the dome antenna. The dielectric constant of the glycerin is about 5 at 6 GHz which is close to that of ABS. The main purpose of using glycerin is to mitigate the friction making the rotation smooth.

### Operation

**Figure 4:**
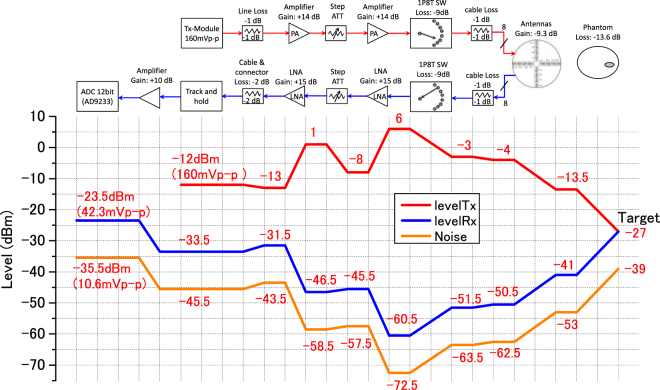
The level diagram of the system for Tx signal, Rx signal and noise.

During the detection, the Gaussian monocycle pulse train at the repetition frequency of 100 MHz is generated by the GMP generator chip. The duration of the pulse is approximately 160 picoseconds. The center frequency of the pulse is 6 GHz and the bandwidth is 6.7 GHz covering the UWB range. The selection of this signal is a trade-off between resolution and penetrating depth. The GMP is transmitted through an amplifier module. The amplitude of the signal is increased to 1.2 volt. Then the antennas are excited in turn. The reflection from the target is received by the other antennas and the Rx SP8T-SW decides which antenna picks up the signal. Then the Rx module samples and digitizes the signal. The level diagram of the system is shown in Fig. 4. The GMP signal with 160 mV_pp_ (−12 dBm) is generated and -4 dBm GMP is transmitted to the antenna through SP8T-SW. Due to the loss in the human breast, the signal arrived at the target is -27 dBm. The received signal is −51.5 dBm at the Rx SP8T-SW so that the signal is amplified up to −23.5dBm (42.3 mV_pp_) at the ADC.

Since it is difficult to sample the signals at the speed of tens of Giga samples per second directly even by the state-of-the-art CMOS technology, the equivalent time sampling method is developed^40^. Since the reflected signal is also periodic at 100 MHz, the received signal can be sampled in one phase (9.77 ps) in one period (10 ns). And then, in the next period after 10 ns plus 9.77 × 2 ps, the next phase is sampled. By sliding the phase of the clock with 9.77 ps, the sampling is conducted like a slow-motion. In this detector, the equivalent sampling speed is 102.4 Giga samples per second. For each signal, there are 2048 measured points in 20 ns and the signal is averaged for 2048 times to reduce the random error.

During the operation, the detector is held by hand as shown in Fig. 1(f). The motor box and housing case are kept stationary. Only the circuit block and the dome antenna array are rotated together, avoiding the coaxial cables being twisted which may result in a large variation of the received signals. Before conducting experiments, the detector is warmed up for 2 hours to make the temperature stable. After the test is started, the step motor rotates and the data are saved automatically until the end. Then, the data are processed offline for breast imaging. The experiment is conducted by rotating in order to extract the target response from the raw data. It takes 7 seconds to collect the data set at one angle. In this report, the rotation is carried out at 3 degree step from 0 to 360 degree. Totally, 120 sets of data are acquired and the total time is 14 minutes. All data are saved and transferred to the laptop computer for the post offline processing. It takes 2 minutes to reconstruct the image using a personal computer with the i7-4790 CPU and 8 GHz memory.

### Imaging

Before image reconstruction, the target signal should be separated from the original received signal since the direct wave and early reflections from the skin compose the dominant part of the received signal and the amplitude of the target reflection is much weaker which is −60.5 dBm after the Rx SP8T-SW as shown in Fig. 4. In this study, the averaging method is utilized to extract the target signal^43^.

Define the entire received signals as

1 $$\,{\bf{S}}=\{{{\bf{S}}}_{1},\,\ldots ,{{\bf{S}}}_{i},\,\ldots ,{{\bf{S}}}_{N}\},$$

where *N* is the number of channel which is the Tx-Rx antenna pair. ${{\bf{S}}}_{i}$ is composed of the signals from channel *i* at all angles as

2 $$\,{{\bf{S}}}_{i}=\{{{S}}_{i1}[n],\,\ldots ,{{S}}_{ij}[n],\,\ldots ,{{S}}_{iM}[n]\},$$

where *j* is the index of the rotation angle, *M* is the total number of the angles rotated and *n* represents the discrete time sample. Because of the jitter existing in the system, the received signals have phase difference between each other which will introduce a big noise residual in the dominant part after subtraction. Therefore, the alignment based on least square method is applied to suppress the phase error which can be depicted as

3 $$\mathop{\min }\limits_{{\rm{\Delta }}{n}_{ij}}\sum _{n={t}_{s}}^{{t}_{e}}{|{S}_{i1}[n]-{S}_{ij}[n+{\rm{\Delta }}{n}_{ij}]|}^{2},$$

where Δ*n*_*ij*_ is the amount of time offset to compensate the phase shift between signals. *t*_*s*_ and *t*_*e*_ are the start time and end time of the range, respectively, for calculating the optimal Δ*n*_*ij*_. The details can be found in ref.^43^. Since the dominant parts of the signals from the same channel are similar, by averaging the signals, a reference signal can be obtained as

4 $${S}_{i,ave}=({S}_{i1}[n]+\cdots +{S}_{iM}[n+{\rm{\Delta }}{n}_{iM}])/M.\,$$

Then the target information can be extracted by subtracting the reference signal from the original ones. The subtracted signal from *i*th channel and *j*th rotation angle is denoted as *D*_*ij*_[*n*]. The finite impulse response (FIR) bandpass filter is applied to the subtracted signals for suppressing noise as

5 $${y}_{ij}[n]=\sum _{k=0}^{l}h[k]\cdot {D}_{ij}[n-k],$$

where *h* is the filter coefficients and *l* is the filter order. The processed data {*y*_*ij*_[*n*]} are used for the image generation.

The DAS method is adopted in our system for its robustness and less computation consuming. For any focal point **r**, the length of the propagation path corresponding to a certain channel can be calculated as

6 $$L({\bf{r}})=|{{\bf{r}}}_{Tx}-{\bf{r}}|+|{{\bf{r}}}_{Rx}-{\bf{r}}|,$$

where **r**_Tx_ and **r**_Rx_ are the positions of the Tx and Rx antenna. The *x* and *y* coordinates of antenna at *j*th rotation angle besides the initial one can be calculated by

7 $$\begin{array}{rcl}{x}_{j} & = & {x}_{center}+\sqrt{{({x}_{1}-{x}_{center})}^{2}+{({y}_{1}-{y}_{center})}^{2}}\\  &  & \cdot \,\cos ({\tan }^{-1}(\frac{{x}_{1}-{x}_{center}}{{y}_{1}-{y}_{center}})-(j-1)\cdot \theta )\\ {y}_{j} & = & {y}_{center}+\sqrt{{({x}_{1}-{x}_{center})}^{2}+{({y}_{1}-{y}_{center})}^{2}}\\  &  & \cdot \,\sin ({\tan }^{-1}(\frac{{x}_{1}-{x}_{center}}{{y}_{1}-{y}_{center}})-(j-1)\cdot \theta ),\end{array}$$

where *x*_center_ and *y*_center_ are the position of the dome center in *x-y* plane. *θ* is the rotation step. Since the rotation is parallel to the *x*-*y* plane, the *z*-coordinate is constant. Then the delay time of the reflection from the selected focal point can be calculated as

8 $${\tau }_{{\bf{r}}}=L({\bf{r}})\cdot \frac{\sqrt{{\varepsilon }_{r}}}{c},$$

where *c* is the speed of light, ${\varepsilon }_{r}$ is the effective permittivity of the breast interior. Here it is assumed to be constant. Finally, the intensity of the focal point *I*(**r**) is calculated by adding the contribution of all the channels and all the rotation angles as

9 $$I({\bf{r}})=\sum _{i=1}^{N}\sum _{j=1}^{M}{y}_{ij}({T}_{c}+{\tau }_{{\bf{r}}}),$$

where *T*_c_ is the system calibration time, which is defined as the timing that the signal is emitted from Tx antenna. Since the sampling timing between experiments changes systematically, all the data set in one experiment will have a common delay time *T*_*c*_. By calculating the intensities of all focal point inside the imaging area, the reflection energy distribution can be obtained. From the imaging results, the position of the target can be estimated.

### Performance Evaluation

Before the evaluation of the performance in detection, the specific absorption rate (SAR) distribution is investigated to ensure the safety of the patient. The SAR is calculated in the CST MICROWAVE STUDIO® simulation environment. During the operation, only one Tx antenna is excited in one switching sequence. Therefore, the output power of the system, which is −13.5 dBm (0.045 mW) as shown in Fig. 4, is transmitted to one antenna illuminating the breast and the SAR is calculated on the average mass of 1 gram. The maximum SAR within the breast is calculated over the UWB frequency band which is from 3.1 to 10.6 GHz. The results are from 0.0118 to 0.0246 W/kg, which are two orders of magnitude lower than the IEEE safety limit of 2 W/kg^44^. These results demonstrate the safety of the detector and that the detector can be applied to clinical trials.

**Figure 5:**
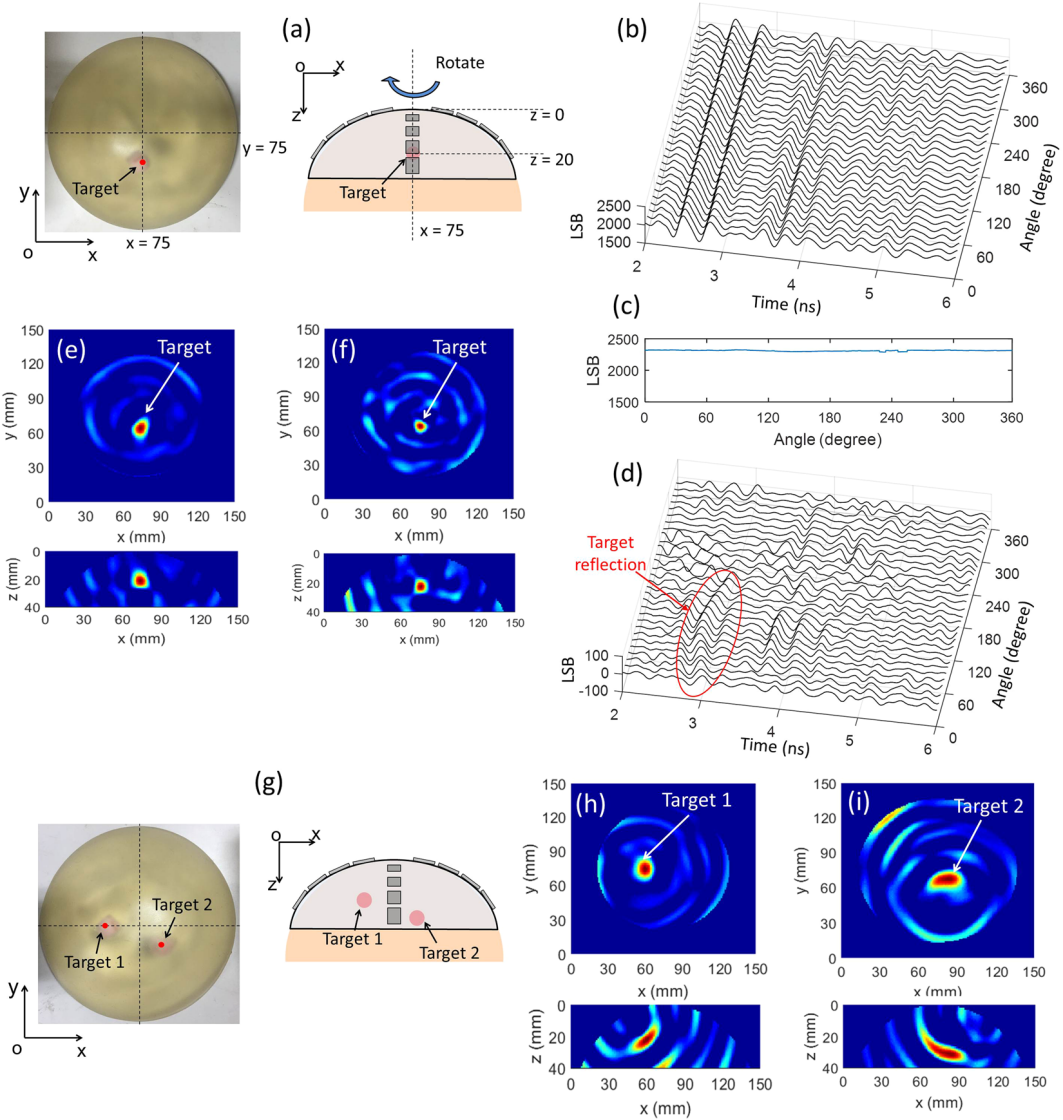
Performance evaluation of breast phantoms. (**a**) The breast phantom with one bacon target inserted showing the target position and the rotation of the antenna array on the breast phantom. (**b**) The three-dimensional plot of the digital received waveforms corresponding to the rotation angles. (**c**) The maximum peak value of the waveforms corresponding to the rotation angles. (**d**) The three-dimensional plot of the subtracted signal waveforms where the target reflections are shown. (**e**) The reconstructed confocal image of the target with the size of 1 cm^3^ in *x-y* plane and *x-z* plane. (**f**) The reconstructed confocal image of the target with the size of 5 × 5 × 5 mm^3^ in *x-y* plane and *x-z* plane. (**g**) The breast phantom inserted with two bacon targets. (**h**) The reconstructed confocal image of the first target in *x-y* plane and *x-z* plane. (**i**) The reconstructed confocal image of the second target in *x-y* plane and *x-z* plane.

In order to confirm the performance of the detector and the imaging method, a set of experiments are conducted by use of the breast phantom. The Silicone breast prosthesis is used as the breast phantom. This prosthesis is originally used for breast reconstruction after mastectomy. The dielectric constant of the silicone is 4 at 6 GHz. The center of the breast phantom is located at (75, 75) in the *x-y* plane, coinciding with the center of antenna array as shown in Fig. 5(a). The zero-point of *z*-axis is defined at the top of the dome antenna and the positive direction is towards the breast depth. The detector rotates in clockwise around the antenna center. First, a 1-cm^3^ bacon target is inserted into the phantom, whose dielectric constant is 45 at 6 GHz. The target position is (75, 60, 20) mm in the *x-y-z* coordinates. The signals are obtained from 120 angles and a part of them are shown in Fig. 5(b). It can be seen that the phase error is eliminated after alignment and the signals are almost the same. The maximum peak of the received signals after analog-to-digital conversion is 2300 LSB (least significant bit) corresponding to the 12-bit ADC. The change of the maximum peak with rotation angle is shown in Fig. 5(c). Since the target reflections are very weak compared with the direct wave, they cannot be observed explicitly in the raw signals. The reference signal is formed by averaging all received signals shown in Fig. 5(b) to eliminate the reflected signals from the target which appear at different arrival times. After subtracting the reference signal, the target reflections can be extracted in the subtracted signals. The subtracted waveforms for 0–360 degree rotation (120 angles) are shown in Fig. 5(d). Note that the amplitude is 100 LSB after analog-to-digital conversion due to the loss in the phantom. Applying the confocal algorithm to the subtracted signals, the breast reconstructed image is generated as shown in Fig. 5(e). From the image, the target is successfully recognized and the estimated position is (74, 64, 21) which agrees with the embedded one. When the target size is 5 × 5 × 5 mm^3^, the corresponding imaging result is shown in Fig. 5(f) and the estimated position is (76, 64, 23) which is in accordance with the real one. The successful detection demonstrates the detection ability of target with the size as small as 5 × 5 × 5 mm^3^. Then, two targets with the size of 1 cm^3^ are inserted into the breast phantom to test the performance in multi-target detection of the system as shown in Fig. 5(g). The position of target one is (60, 75, 20) and the other one is (85, 65, 30) in *x-y-z* coordinates. The progressive processing method is utilized to detect the targets respectively by the antenna selection^45^. The results are shown in Fig. 5(h) and Fig. 5(i). The estimated positions of the targets are (60, 75, 22) and (84, 68, 31), which are consistent with the embedded position. Both targets are successfully recognized, demonstrating the feasibility of system on multi-target detection.

**Figure 6:**
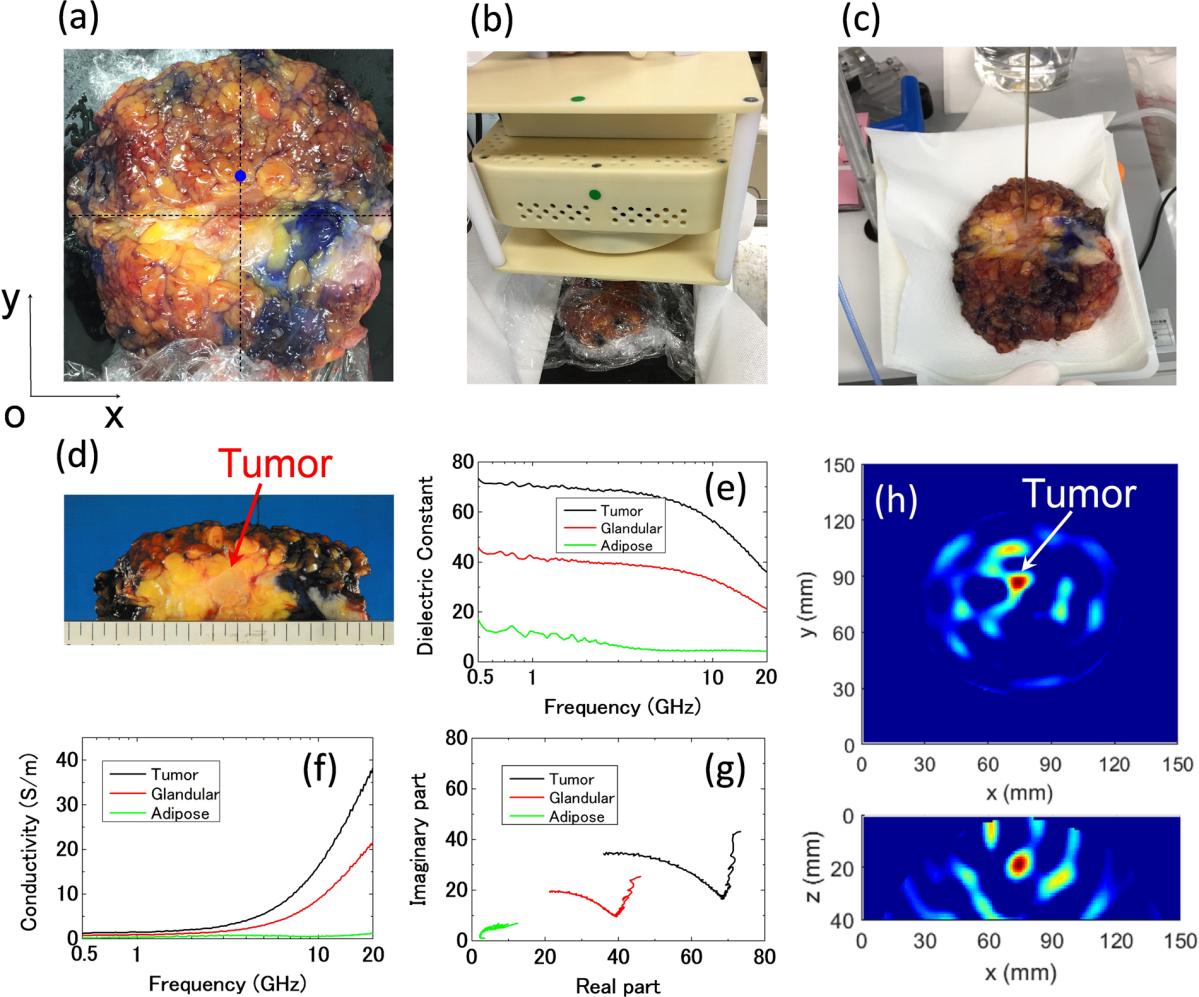
Performance evaluation of the excised breast after mastectomy. (**a**) The breast after dissection by a pathologist. (**b**) The hand-held detector on the excised breast being covered with a poly-ethylene film. (**c**) The measurement of the electromagnetic properties of the breast tissues by a 2.2-mm-diameter coaxial probe. (**d**) The cross-section photograph of the breast tissues. (**e**) The dielectric constants of the tumor, glandular and adipose tissues. (**f**) The conductivities of the tumor, glandular and adipose tissues. (**g**) The Cole-Cole plot of the breast tissues. (**h**) The reconstructed imaging results of the breast of *x-y* and *x-z* cross sections.

Next, the system is applied to excised breast tissues after total mastectomy surgery at Hiroshima University Hospital. The experiment procedure is as follows. After the mastectomy surgery operation is over, the breast is first dissected by the pathologist doctor to confirm the position of the tumor and take tumor samples. Then the breast is covered with poly ethylene film to isolate the blood and fold the cut incision. The breast is positioned carefully, keeping the incision along with the *x* axis and the tumor on the *x* = 75 line as shown in Fig. 6(a). Then, the detector is placed on the excised breast to conduct the detection experiment as shown in Fig. 6(b). After the experiment, the electrical properties of the tissues are measured using a 2.2-mm-radius coaxial probe following the procedures presented in ref.^1^ as shown in Fig. 6(c). Dielectric constants and conductivity versus frequency for tumor, glandular and adipose tissues are shown in Fig. 6(e)-(f). Cole-Cole plot for the breast tissues is shown in Fig. 6(g). The measurement results show that the difference between tumor tissue and normal tissues are apparent. The imaging result is shown in Fig. 6(h). From the reconstructed breast image, the tumor is successfully recognized and the position is almost consistent with the tumor physical position. Compared with the phantom experiment results, the signal-to-noise ratio becomes worse in the excised breast case. This is caused by the inhomogeneous feature of the real breast tissues. The detectability of malignant tumors is demonstrated in excised breast tissues after mastectomy surgery. The three-dimensional positions of the tumors are consistent with the results of the histopathology analysis. The successful detection of the tumor in the excised breast demonstrates the robustness of the system and the processing algorithm.

### Pilot clinical test

In the clinical applications, five volunteer patients, who have been diagnosed to have breast cancers, were recruited with ethical approval and informed consent. The malignant tumors include invasive ductal carcinoma (IDC) and ductal carcinoma *in situ* (DCIS). The patients were informed the total process of the detection and signed the consent. Before the test, the clinical doctors were trained how to use the detector. The clinical tests were conducted at the Hiroshima University Hospital by the oncologists without any engineers on the spot. After the tests, the raw data were sent and the information of the cancer details was not notified. Then, the imaging process was conducted and the results were sent to the doctors for evaluation. Finally, by comparing the imaging results with the MRI, X-ray and PET (position emission tomography), the conclusion was made.

**Figure 7:**
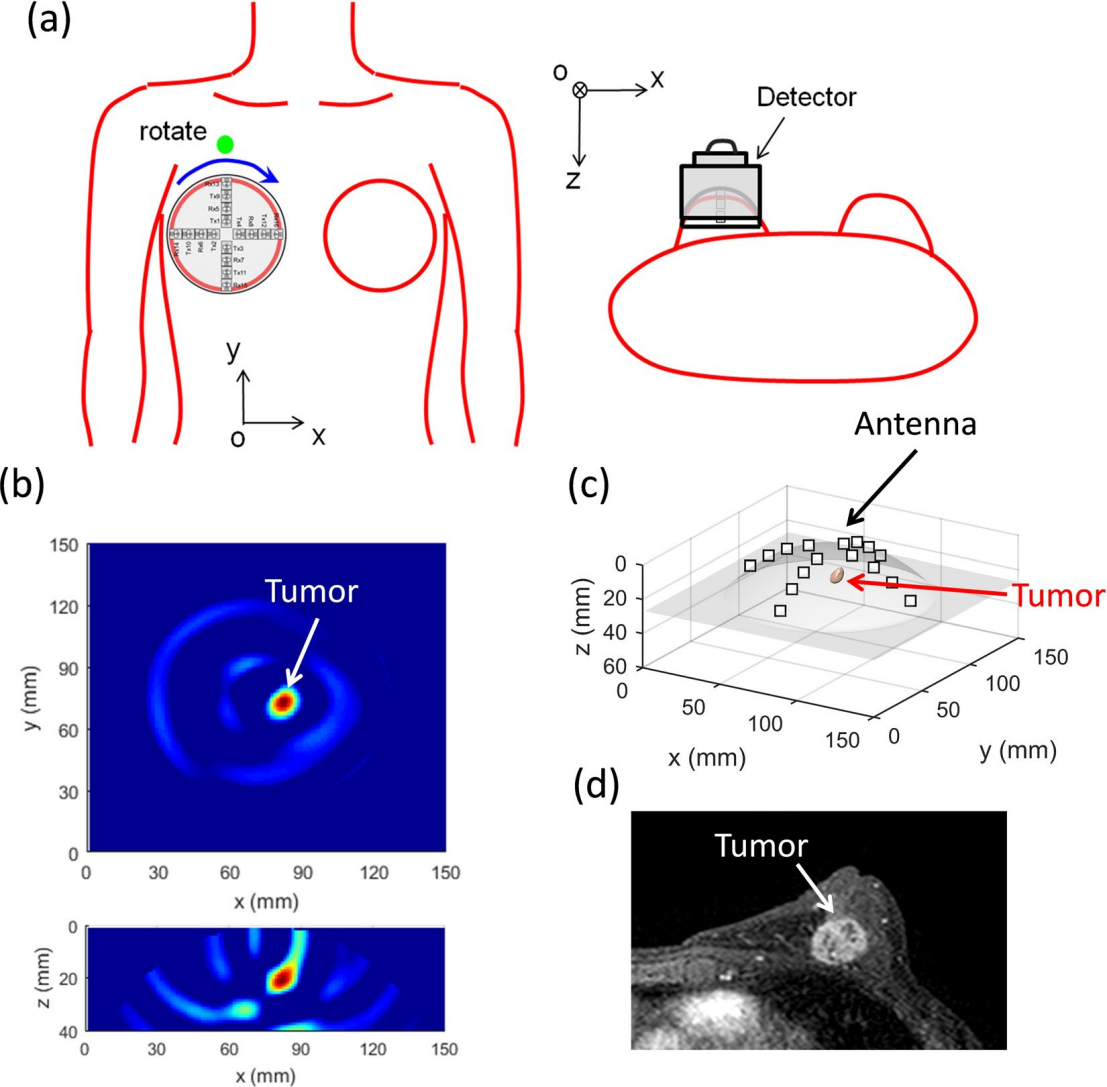
Pilot clinical test. (**a**) Configuration of the clinical test for the right breast with a supine position. (**b**) The reconstructed confocal imaging results of *x-y* and *x-z* cross sections. (**c**) The reconstructed 3D display of the imaging area showing the location of the tumor with respect to the antenna array. (**d**) The MRI scan of the breast.

During the test, the patients were asked to lie in the supine position and the detector is put on the breast as shown in Fig. 7(a). There is a green marker pasted on the outside of the system, indicating the position of the start angle and pointing to the head. The rotation is clockwise. The x-axis is set on the intersection between the transverse plane and coronal plane. The y-axis is set on the intersection between the sagittal plane and coronal plane.

The confocal imaging results of one patient breast scan are shown in Fig. 7(b). The 3D display area as shown in Fig. 7(c) is created by thresholding at 0.7 of the maximum. The grey cover is the profile of the dome antenna and the white squares are the antenna positions. The MRI scan is shown in Fig. 7(d). There is a good agreement in both methods, demonstrating the detectability of the breast tumor using the breast cancer detector.

### Ethical approval and informed consent

The performed pilot clinical tests are conducted following the guidelines and protocols of the Japan Clinical Oncology Group protocol manual ver.2.6, which approved the breast cancer detector and its measurement procedure (Hiroshima University Clearance number: C-153). The UMIN-CTR (University Hospital Medical Information Network-Clinical Trials Registry) number is UMIN000026181. The informed consents of all volunteers are obtained before the clinical test.

## Conclusion

The detectability of a hand-held impulse-radar breast tumor detector is demonstrated. The successful detection of the tumor in the excised breast after mastectomy shows the feasibility of the hand-held impulse-radar detector system in real inhomogeneous scenario. The pilot clinical test is conducted on malignant breast tumors of IDC and DCIS for five patients at the hospital. The imaging results show a good agreement with those of MRI. These promising results show the possibility of hand-held breast tumor detection and monitoring system for easy access to everyone.
